# Effects of Different Drying Methods on the Characterization, Dissolution Rate and Antioxidant Activity of Ursolic Acid-Loaded Chitosan Nanoparticles

**DOI:** 10.3390/foods10102470

**Published:** 2021-10-15

**Authors:** Lujie Zhao, Xu Duan, Weiwei Cao, Xing Ren, Guangyue Ren, Panpan Liu, Junliang Chen

**Affiliations:** College of Food and Bioengineering, Henan University of Science and Technology, Luoyang 471023, China; ZLujie942658@163.com (L.Z.); caoweiwei@haust.edu.cn (W.C.); rennxing@163.com (X.R.); guangyueyao@163.com (G.R.); liupanpan1002@163.com (P.L.); chenjl2020@haust.edu.cn (J.C.)

**Keywords:** ursolic acid, chitosan nanoparticles, microwave freeze drying, dissolution study

## Abstract

To improve the water solubility of ursolic acid (UA), UA-loaded chitosan nanoparticles were firstly prepared by the ionotropic gelation method and dried by freeze drying (FD), microwave freeze drying (MFD) and spray drying (SD). The characterization of UA-loaded chitosan nanoparticles was performed with particle size, drug loading (DL), scanning electron microscope (SEM), fourier transform infrared spectroscopy (FT-IR), differential scanning calorimetry (DSC), dissolution studies and antioxidant activity. The results demonstrated that UA was successfully encapsulated into chitosan nanoparticles using sodium tripolyphosphate (TPP) as a cross-linker, with a 79% encapsulation efficiency. The spray-dried, UA-loaded chitosan nanoparticles had the lowest drug loading (11.8%) and the highest particle size (496.9 ± 11.20 nm). The particle size of UA-loaded chitosan nanoparticles dried by MFD and FD was lower, at 240.8 ± 12.10 nm and 184.4 ± 10.62 nm, respectively, and their antioxidant activity was higher than those nanoparticles dried by SD. Moreover, the drying time and energy consumption of UA-loaded chitosan nanoparticles dried by MFD and SD were lower than that of FD. The dissolution rates of UA-loaded chitosan nanoparticles prepared by FD and MFD were 60.6% and 57.1%, respectively, in a simulated gastric fluid, which was a greater value than SD (55.9%). Therefore, the UA-loaded chitosan nanoparticles encapsulation method, combined with MFD technology, showed a promising potential to improve the water solubility of UA.

## 1. Introduction

Ursolic acid (UA) is a representative small-molecule compound of pentacyclic triterpenoid found in Fructus Corni [1,2] and exhibits various biological activities, such as anti-inflammatory, antioxidant and anti-diabetic effects [3,4]. In addition, UA also features a low toxicity and an easier availability in nature [5]. However, UA is not broadly used in health products and cosmetics because of its poor water solubility and low bioactivity [6,7]. Therefore, it is necessary to explore suitable approaches to overcome the low water solubility of UA.

A number of delivery systems have been developed to enhance UA solubility, including solid inclusion complexes, liposomes, nano-emulsions and hydrogels. Nowadays, natural polymer nanoparticles are a research hotspot due to their distinct advantages such as their small size and effective penetration ability [8,9]. Chitosan, as a natural polymer, is a linear and naturally cationic polymer obtained by the partial deacetylation of chitin [10]. Chitosan-based nanoparticles are widely used as nano-carriers for delivering active ingredients to increase stability and bioavailability [11]. For example, chitosan and alginate nanocapsules were prepared by the solvent displacement technique to improve its solubility of curcumin [12]. The resveratrol loaded zein/chitosan colloidal particles prepared by the classical antisolvent procedure had a higher stability and bioavailability [13]. Previous studies on UA-loaded chitosan focused on organic, acid-modified chitosan nanoparticles. Chitosan modified poly (lactic acid) nanoparticles by the emulsion–solvent evaporation method, which was used extensively to improve the bioavailability of UA [14]. The folate–chitosan nanoparticles loaded with UA could increase the solubility of UA [15]. Zhao et al. [16] successfully encapsulated UA in the mesoporous silica nanoparticle chitosan-lactobionic acid (MSN-CS-LA) nanocarrier system through non-covalent interactions. Although the above organic acid-modified chitosan nanoparticles can significantly improve the solubility and bioavailability of UA, they have the disadvantages of redundant procedures, a larger particle size and a low drug-loading efficiency. The reaction of chitosan and sodium tripolyphosphate (TPP) without organic acid modification can easily form nanoparticles via electrostatic interaction. However, studies on the UA chitosan-TPP nanoparticles by the ionotropic gelation method have not yet been performed. Therefore, the preparation of UA-loaded chitosan-TPP nanoparticles to encapsulate UA was investigated.

Drying is a necessary unit operation to obtain chitosan nanoparticles. Common techniques in the fabrication of chitosan nanoparticles are the spray drying (SD) and freeze drying (FD) [17]. SD was applied in preparing the amoxicillin chitosan nanoparticles and erlotinib chitosan nanoparticles [18,19]. However, the inlet temperature during the SD process was above 100 °C and could cause the degradation of sensitive compounds [20]. FD produces high-quality nanoparticles with an excellent retention ratio and a minimal thermal and long-term storage stability [21,22,23], but always requires more time and energy consumption to obtain dry products. In the current research, UA-loaded nanoparticle powder is generally obtained by FD [24,25,26], but the drawbacks of a longer duration and higher cost exist in the FD process of UA nanoparticles. In order to avoid the disadvantages of SD and FD, microwave freeze drying (MFD) as a novel drying technology can be used to produce high-quality products with less energy consumption [27,28]. Duan et al. [29] reported that the time for drying a sea cucumber by MFD was nearly half of that for FD, and there were no significant differences in the quality of the microwave freeze-dried and standard freeze-dried sea cucumbers. Moreover, MFD could realize a better dry mushroom quality and drying efficiency than FD [30]. Therefore, as a low-energy method, MFD is a potential drying technology to obtain nanoparticles in high quality. We hypothesized that MFD for drying UA nanoparticles could achieve a better retention of UA at a lower cost. However, there are no studies about the preparation of UA-loaded chitosan nanoparticles by MFD.

In this research, the ionotropic gelation method was used to fabricate UA-loaded chitosan nanoparticles, and the nanoparticle powders were dried by FD, MFD and SD, respectively. The UA-loaded chitosan nanoparticles were characterized in terms of encapsulation efficiency (EE), scanning electron microscope (SEM), fourier transform infrared spectroscopy (FT-IR), differential scanning calorimetry (DSC), dissolution studies and antioxidant activity. The findings of this research might broaden the methods of fabricating UA nanoparticles and contribute to the application of UA in functional food.

## 2. Materials and Methods

### 2.1. Materials

Chitosan (degree of deacetylation ≥90.0%), with a molecular weight of 30,000, was purchased from Shanghai Lanji Technology Development Co., Ltd. (Shanghai, China). UA (purity: 98%) was purchased from Chengdu Prefa Technology Development Co., Ltd. (Chengdu, Sichuan, China). TPP and Tween-80 were analytical grade reagents purchased from Tianjin Deen Chemical Reagent Co., Ltd. (Tianjin, China). The remaining chemical reagents were of analytical grade.

### 2.2. Preparation of UA-loaded Chitosan Nanoparticles

UA-loaded chitosan nanoparticles were prepared according to the method of Yan et al. [31] and optimized. Chitosan was added to 1% (*w/v*) acetic acid solution for magnetic stirring for 8 h at 25 °C to obtain transparent solution. The pH of chitosan solution was adjusted to 5.0 with 0.1 g/mL sodium hydroxide solution, and then 1% Tween-80 was added and magnetically stirred for 20 min at 25 °C. After that, the UA solution dissolved in ethanol was added to the above solution at the UA–chitosan mass ratio of 4:1, and under magnetic stirring for 30 min. TPP solution of 2.0 mg/mL was slowly dropped into the mixed solution at the chitosan-TPP mass ratio of 4:1, and magnetically stirred for 45 min to obtain UA-loaded chitosan nanoparticles suspension. The nanoparticles suspension was centrifuged at 12,000 rpm for 20 min, and the precipitate was washed with distilled water to remove unbound UA, and dried for further studies.

### 2.3. Drying Experiments

#### 2.3.1. Spray Drying (SD)

The materials were dried by a spray dryer (YC-015, Pilotech Instrument & Equipment Co., Ltd., Shanghai, China). The UA-loaded chitosan nanoparticles suspension was fed into the chamber through a peristaltic pump at a feed flow rate (300 mL/h). The inlet temperature, outlet temperature and air flow rate were 120 °C, 80 °C and 1.3 m^3^/min, respectively. The spray-dried, UA-loaded chitosan nanoparticles were collected and stored in desiccators until analysis.

#### 2.3.2. Freeze Drying (FD)

The precipitate was dried by a vacuum freeze dryer (LGJ-10D, Beijing Science Instrument Co. Ltd., Beijing, China). The precipitate was placed into Petri dish and frozen at −25 °C for at least 8 h. The frozen precipitate was put into the drying chamber at the pressure of 40 Pa. The cold trap and heat shelf temperatures were set at −40 °C and −50 °C, respectively. The precipitate was frozen in the vacuum freeze dryer for about 6 h. The freeze-dried, UA-loaded chitosan nanoparticles were stored in desiccators until analysis.

#### 2.3.3. Microwave Freeze Drying (MFD)

The precipitate was dried by a microwave freeze dryer which was developed by Duan et al. [32]. The precipitate was frozen at −25 °C for at least 8 h. The pressure of the independent polypropylene drying cavity and cold trap temperature was carried out at 100 Pa and −40 °C, respectively. The power of microwave was set at 20 W. The microwave freeze-dried, UA-loaded chitosan nanoparticles powders were stored in desiccators until analysis.

### 2.4. Characterization of UA-Loaded Chitosan Nanoparticles

#### Encapsulation Efficiency (EE) and Drug Loading (DL)

After UA-loaded chitosan nanoparticles were prepared according to 2.2, the UA nanoparticle suspension was centrifuged at 10,000 rpm for 20 min. The supernatant was separated and the precipitate was washed with distilled water. Ethanol was added to the precipitate and sonicated for 15 min, centrifuged at 10,000 rpm for 15 min, the absorbance at 210 nm was analyzed by using UV spectrophotometer (UV-2600, Shanghai Ronnik Instrument Co. Ltd., Shanghai, China), and the content of UA was calculated by the standard curve. The EE and DL were calculated using the following Equations (1) and (2), respectively [33]:

(1) $$\mathrm{EE}\;(\%)=\frac{\mathrm{amount}\;\mathrm{of}\;\mathrm{encapsulated}\;\mathrm{UA}\;\mathrm{in}\;\mathrm{nanoparticles}}{\mathrm{amount}\;\mathrm{of}\;\mathrm{UA}\;\mathrm{initially}\;\mathrm{added}}\times 100$$

(2) $$\mathrm{DL}\;(\%)=\frac{\mathrm{amount}\;\mathrm{of}\;\mathrm{encapsulated}\;\mathrm{UA}\;\mathrm{in}\;\mathrm{nanoparticles}}{\mathrm{weight}\;\mathrm{of}\;\mathrm{UA}\;\mathrm{chitosan}\;\mathrm{nanoparticles}}\times 100$$

### 2.5. Particle Size and Polydispersity Index (PDI)

The particle size and PDI of the UA-loaded chitosan nanoparticles dried by different methods were measured by using a dynamic light scattering technique (Zetasizer model Nano ZS, Malvern Instruments, Malvern, UK) [34]. All the samples were measured in triplicates.

### 2.6. Scanning Electron Microscope (SEM)

The UA-loaded chitosan nanoparticles were sprinkled on the double-sided adhesive tape and coated with gold [35]. The microstructure and surface morphology of UA-loaded chitosan nanoparticles were observed with SEM (TM3030Plus, Hitachi High-Tech Corporation, Tokyo, Japan) at magnification 20,000×.

### 2.7. Fourier Transform Infrared (FT-IR) Spectroscopy

FT-IR spectrophotometer (VERTEX70, German BRUKER Company, Karlsruhe, German) was used to analyze the UA-loaded chitosan nanoparticles. The spectra were recorded in the scanning range of 4000–400 cm^−1^ at a resolution of 4 cm^−1^ [36].

### 2.8. Differential Scanning Colorimetry (DSC)

DSC was used to analyze the effect of different drying methods on the thermal behavior of UA-loaded chitosan nanoparticles. The powders were evaluated using DSC (Switzerland METTLER-TOLEDO, Zurich, Switzerland). Approximately 5 to 10 mg of samples were weighted and set in hermetically sealed aluminum pans and the cover lid was poked. DSC analysis was heated from 50 °C to 400 °C and the heating rate was 10 °C/min. Nitrogen was used as the purge gas at a constant flow rate of 100 mL/min. An empty hermetically sealed aluminum pan was used as a reference [37].

### 2.9. Dissolution Study

The UA-loaded chitosan nanoparticles were added to a beaker containing simulated gastric fluid (SGF, pH 2.0, 0.01 mol/L hydrochloric acid and 0.09 mol/L sodium chloride) and simulated intestinal fluid (SIF, pH 6.9, 0.07 mol/L potassium dihydrogen phosphate and 0.2 mol/L sodium hydroxide), and stirred at 120 rpm at 37 °C. Suspensions were sampled at appropriate time intervals and replaced with same volume of fresh dissolution medium to maintain the sink conditions. The withdraw samples were immediately filtered through 0.45 μm filter membrane and analyzed by UV [38,39].

### 2.10. Antioxidant Activity

Antioxidant activity of UA-loaded chitosan nanoparticles was measured using DPPH free radical scavenging capacity. DPPH (4 mg) were dissolved in 100 mL ethanol solution to obtain DPPH solution. The UA-loaded chitosan nanoparticles were dissolved in ethanol solution and mixed with DPPH solution. After 30 min, the absorbance was measured at 517 nm. The results were calculated according to the vitamin C (V_C_) standard curve and Equation (3). The equation of DPPH scavenging activity was as follows:

(3) $$\mathrm{DPPH}\;\mathrm{scavenging}\;\mathrm{activity}\;\left(\mathrm{mg}\;\mathrm{Vc}/g\right)=\left(\;\frac{1-\mathrm{As}}{\mathrm{Ao}}\;\right)\times 100$$

where, A_s_ is the absorbance of the sample mixed with ethanol solution of DPPH and A_o_ is the absorbance of DPPH solution.

### 2.11. Statistical Analysis

The results were performed as means ± SD. Origin 2017 software was used to draw results diagrams. In order to determine the significant difference between the group samples, the confidence interval was selected as 95% (*p* < 0.05).

## 3. Results and Discussion

### 3.1. Particle Size, PDI and Morphology of UA-Loaded Chitosan Nanoparticles

The characteristics of the UA-loaded chitosan nanoparticles dried by different methods are shown in Table 1. The EE of the UA-loaded chitosan nanoparticles was approximately 79%. Table 1 shows that the DL of the UA nanoparticles dried by FD, MFD and SD were 12.7%, 12.0% and 11.8%, respectively, indicating that the DL of spray-dried nanoparticles was lower than microwave freeze-dried and standard freeze-dried nanoparticles. This may be due to the high inlet temperature of the SD process causing the degradation of partial UA. Table 1 shows that the drying times of FD, MFD and SD were 24 h, 2 h and 3 h, respectively, which demonstrated that MFD had the shortest drying time and the highest drying efficiency. As shown in Table 1, the particle size of the UA nanoparticles dried by different methods ranged from 190 nm to 531 nm, and the order was as follows: SD > MFD > FD. The PDI of standard freeze-, microwave freeze-, and spray-dried, UA-loaded chitosan nanoparticles were 0.186, 0.515 and 0.476, respectively, which indicated that FD formed more homogeneous populations, compared with MFD and SD [40].

**Figure 1:**
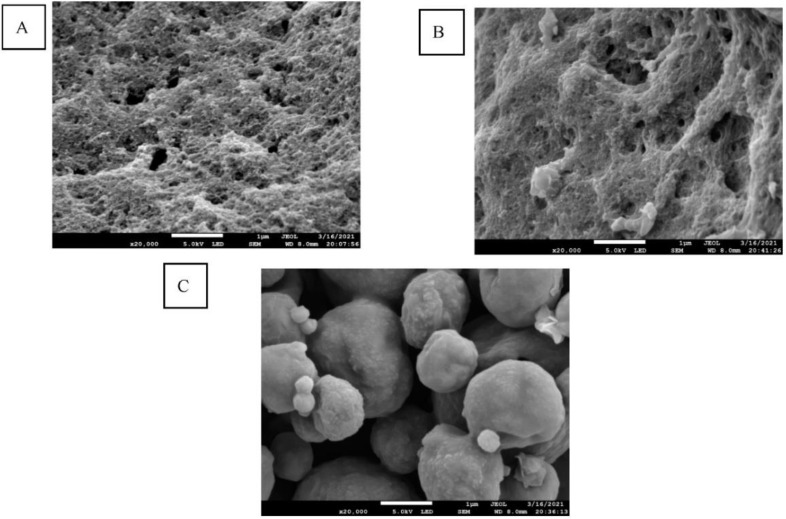
SEM micrographs of UA-loaded chitosan nanoparticles prepared by FD (**A**), MFD (**B**), and SD (**C**).

The morphology of the UA-loaded chitosan nanoparticles dried by three drying methods is shown in Figure 1, where the shape of the dried, UA-loaded chitosan nanoparticles varied significantly depending on the drying method used. Specifically, the morphologies of freeze- and spray-dried nanoparticles were agglomerated small spheres with porous structures (Figure 1A), and spherical types of different sizes (Figure 1C), respectively. Compared with FD, the morphology of the microwave freeze-dried UA nanoparticles (Figure 1B) presented looser porous structures, and the surface of the powders appeared rough due to the effect of microwaves during the drying process.

### 3.2. FT-IR Analysis

**Figure 2:**
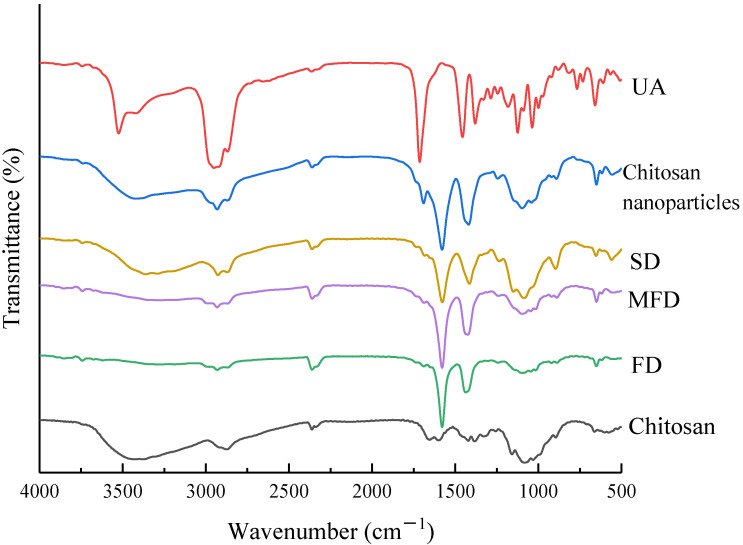
FT-IR spectra of chitosan, UA, chitosan nanoparticles, freeze-, microwave freeze-, and spray-dried UA-loaded chitosan nanoparticles. UA-loaded chitosan nanoparticles prepared by freeze drying (FD), microwave freeze drying (MFD) and spray drying (SD). UA: ursolic acid.

The FT-IR spectra of chitosan, UA, chitosan nanoparticles, and UA-loaded chitosan nanoparticles obtained by different drying methods are illustrated in Figure 2. The spectra of chitosan showed a peak at 3432 cm^−1^ assigned to the O-H stretching vibration. In the spectra of UA, the peaks at 3524 cm^−1^, 1714 cm^−1^ and 1125 cm^−1^ were attributed to the cyclitols’ O-H stretch, the C = C stretching vibration and the cyclitols’ C-O stretch, respectively [41]. The spectra characteristics of chitosan and UA were similar to those reported in previous studies [42,43]. For the blank chitosan nanoparticles, the peak of O-H bending shifted from 3381 to 3432 cm^−1^, and the band became much broader, indicating that the hydrogen bond interaction of chitosan was enhanced and that there was a presence of electrostatic interactions. During the formation of chitosan nanoparticles, the peak shifted from 1653 cm^−1^ and 1599 cm^−1^ in the chitosan spectra to 1578 cm^−1^, suggesting that C = O amide I and the –NH_2_ groups of chitosan were both involved in electrostatic interactions [44]. Besides, the peaks in the range of 780 to 540 cm^−1^ indicated the symmetrical stretching of these bonds, proving the existence of the interaction between chitosan and TPP [42]. The difference in the peak position of 3650 to 3000 cm^−1^ of UA-loaded chitosan nanoparticles under different drying methods was attributed to the discrepancy in the stretching vibration of the O-H. Compared with FD and MFD, the peak of O-H stretching vibration shifted from 3385 cm^−1^ to 3362 cm^−1^ in spray-dried, UA-loaded chitosan nanoparticles, indicating that the intramolecular hydrogen bond strength increased. Compared with the UA FT-IR spectra, the characteristic peaks of all the UA-loaded chitosan nanoparticles dried by the three methods at 1714 cm^−1^ and 1125 cm^−1^ disappeared, which confirmed no chemical interaction between UA and chitosan nanoparticles. Furthermore, the disappearance of the UA peaks indicated the encapsulation of UA into the nanoparticles.

### 3.3. DSC Analysis

**Figure 3:**
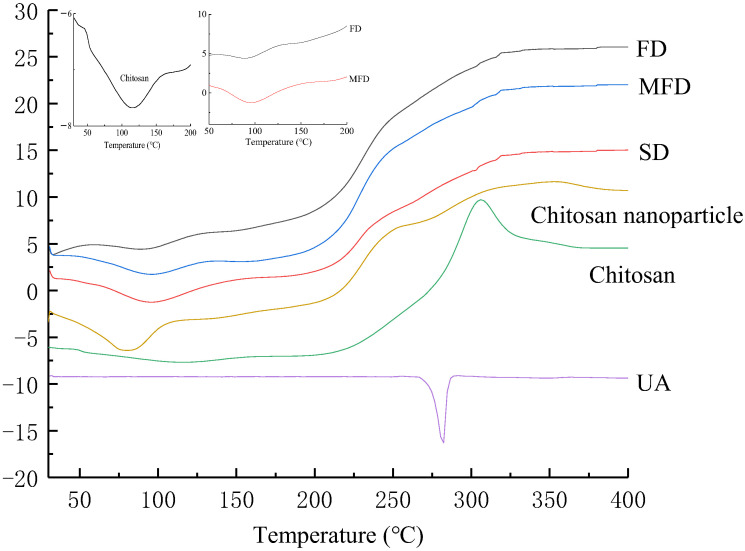
DSC spectra of chitosan, UA, freeze-, microwave freeze- and spray-dried, UA-loaded chitosan nanoparticles. UA-loaded chitosan nanoparticles prepared by freeze drying (FD), microwave freeze drying (MFD) and spray drying (SD). UA: ursolic acid.

Figure 3 shows the DSC spectra of UA, chitosan alone, the chitosan nanoparticle, and the UA-loaded chitosan nanoparticles dried by SD, FD and MFD, respectively. The DSC curve of chitosan showed an endothermic peak near 100 °C and an exothermic peak around 290 °C, respectively, which were related to the loss from the hydrophilic groups of chitosan and structural decomposition, respectively [45]. The DSC curve of the blank chitosan nanoparticle showed an endothermic peak near 90 °C. The variation of the endothermic peak in the chitosan nanoparticle was considered to be the physical and molecular changes caused by the electrostatic interaction between chitosan and TPP [46]. The DSC curve of UA showed an endothermic single peak near 283 °C in Figure 3, which was the melting point of UA, owing to its crystal structure. UA-loaded chitosan nanoparticles dried by SD, FD and MFD showed the decreased in the endothermic peak at 92 to 96 °C compared to chitosan at 100 °C. After UA was encapsulated by the chitosan nanoparticles, the crystalline peak at 283 °C in UA disappeared. These results proved that UA was encapsulated into the chitosan nanoparticles dried by the three methods.

### 3.4. Dissolution Studies

**Figure 4:**
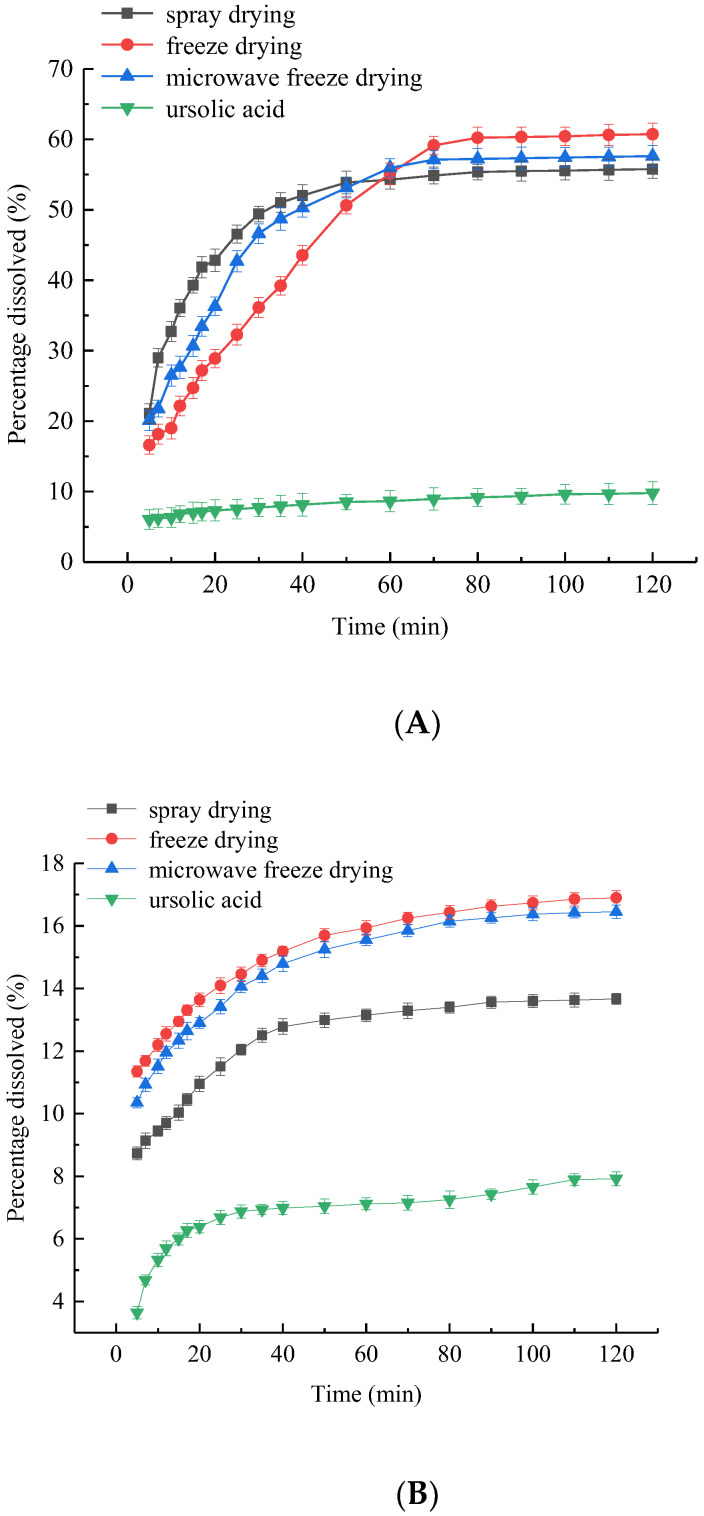
Dissolution curves of UA-loaded chitosan nanoparticles prepared by different methods in SGF (**A**) and SIF (**B**).

Figure 4 displays the dissolution curves of UA-loaded chitosan nanoparticles prepared by different methods in SGF and SIF, respectively. It can be seen from Figure 4 that the dissolution rates of the unencapsulated UA in SGF and SIF were both lower than 10%. Figure 4A showed that, during the first 50 min, UA-loaded chitosan nanoparticles prepared by different drying methods presented a fast dissolution rate in SGF followed by a slight and steady dissolution rate, and the dissolution rate at 50 min was in the order of SD (53.9%) > MFD (53.1%) > FD (50.6%). From 50 to 120 min in SGF, the cumulative dissolution rate of UA-loaded chitosan nanoparticles dried by SD, FD and MFD were 2%, 10% and 4%, respectively. The dissolution rate in SIF of the UA-loaded chitosan nanoparticles prepared by different drying methods was in the order of FD (16.9%) > MFD (16.4%) > SD (13.7%) (Figure 4B). Thus, the UA-loaded chitosan nanoparticles prepared by FD and MFD showed more advantages in increasing the dissolution rate of UA than SD.

### 3.5. Antioxidant Activity

**Figure 5:**
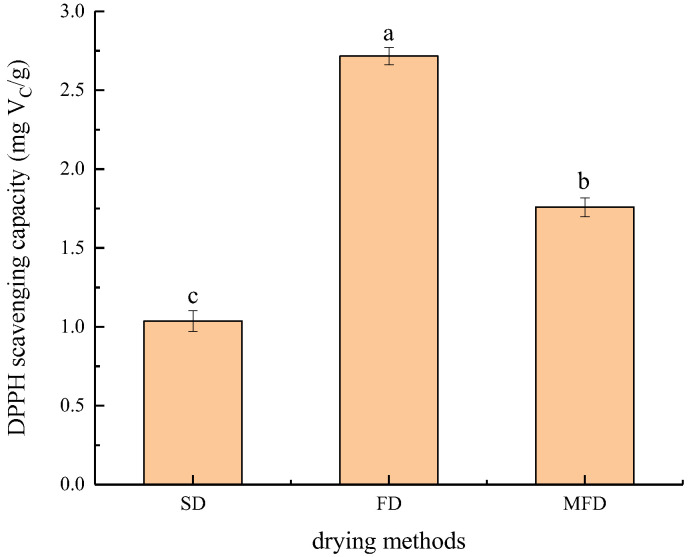
Antioxidant activity of UA-loaded chitosan nanoparticles dried by different methods. ^a–c^ Values with different superscript letters in the figure are significantly different (*p* < 0.05) on the basis of Tukey’s multiple-comparison test.

The DPPH free radical scavenging capacity of UA-loaded chitosan nanoparticles prepared by different drying methods is shown in Figure 5. It was suggested that UA-loaded chitosan nanoparticles prepared by different drying methods exhibited different antioxidant activities. The order of the DPPH free radical scavenging capacity of the UA-loaded chitosan nanoparticles dried by the three drying methods was as follows: FD (2.72 mg V_C_/g) > MFD (1.76 mg V_C_/g) > SD (1.04 mg V_C_/g). These results indicated that the UA-loaded chitosan nanoparticles prepared by FD and MFD had a higher antioxidant activity than that dried by SD, which was attributed to the lower drying temperature of FD and MFD. Therefore, MFD as a new drying technology and FD could be applied in drying UA-loaded chitosan nanoparticles to maintain the biological activity of UA.

## 4. Conclusions

This study demonstrated that UA encapsulated in chitosan-TPP nanoparticles dried by MFD, FD and SD could increase the water solubility of UA. The FT-IR and DSC spectra confirmed the presence of UA in the chitosan nanoparticles. The SEM results confirmed that the UA-loaded chitosan nanoparticles prepared by the three drying methods were in the nano size range (184.4–496.9 nm), and displayed a spherical type in different sizes. UA-loaded chitosan nanoparticles dried by FD and MFD showed a higher antioxidant activity and higher dissolution rates than that of the spray-dried nanoparticles. Among the three drying methods, MFD is a novel and promising technology with the shortest drying time, which can be applied in drying UA-loaded chitosan nanoparticles to increase UA solubility. Therefore, this study provides more references for the application of UA-loaded, chitosan-TPP nanoparticles in functional food to further enhance UA bioavailability.

## Figures and Tables

**Table 1:** Characteristics of the UA-loaded chitosan nanoparticles.

	DL (%)	Drying Time (h)	Particle Size (nm)	PDI
FD	12.7 ± 0.3 ^a^	24	184.4 ± 10.62 ^a^	0.186 ± 0.04 ^a^
MFD	12.0 ± 0.5 ^a^	2	240.8 ± 12.10 ^b^	0.515 ± 0.01 ^c^
SD	11.8 ± 0.2 ^b^	3	496.9 ± 11.20 ^c^	0.476 ± 0.03 ^b^

Values are given as mean ± standard deviation. ^a–c^ Values with different superscript letters in the same column are significantly different (*p* < 0.05) on the basis of Tukey’s multiple-comparison test.

## Abbreviations

DL: drug loading, DSC: differential scanning calorimetry, FD: freeze drying, FT-IR: fourier transform infrared spectroscopy, MFD: microwave freeze drying, SD: spray drying, SEM: scanning electron microscope, TPP: sodium tripolyphosphate, UA: ursolic acid.
